# FSW Optimization: Prediction Using Polynomial Regression and Optimization with Hill-Climbing Method

**DOI:** 10.3390/ma18020448

**Published:** 2025-01-19

**Authors:** Piotr Myśliwiec, Paulina Szawara, Andrzej Kubit, Marek Zwolak, Robert Ostrowski, Hamed Aghajani Derazkola, Wojciech Jurczak

**Affiliations:** 1 Department of Materials Forming and Processing, Rzeszow University of Technology, al. Powst. Warszawy 8, 35-959 Rzeszów, Poland; m.zwolak@prz.edu.pl (M.Z.); rostrows@prz.edu.pl (R.O.); 2 Doctoral School of Engineering and Technical Sciences, Rzeszow University of Technology, al. Powst. Warszawy 12, 35-959 Rzeszów, Poland; p.szawara@prz.edu.pl; 3 Department of Manufacturing and Production Engineering, Rzeszow University of Technology, al. Powst. Warszawy 8, 35-959 Rzeszów, Poland; a.kubit@prz.edu.pl; 4 Department of Nonlinear Solid Mechanics, Faculty of Engineering Technology, University of Twente, 7500-7549 Enschede, The Netherlands; h.aghajaniderazkola@utwente.nl; 5 Mechanical and Electrical Engineering Department, Polish Naval Academy, 81-103 Gdynia, Poland; w.jurczak@amw.gdynia.pl

**Keywords:** friction stir welding, polynomial regression, hill-climbing, response surface analysis, Design Expert 12, welding parameter optimization, aluminum alloy 2024-T3

## Abstract

This study presents the optimization of the friction stir welding (FSW) process using polynomial regression to predict the maximum tensile load (MTL) of welded joints. The experimental design included varying spindle speeds from 600 to 2200 rpm and welding speeds from 100 to 350 mm/min over 28 experimental points. The resulting MTL values ranged from 1912 to 15,336 N. A fifth-degree polynomial regression model was developed to fit the experimental data. Diagnostic tests, including the Shapiro–Wilk test and kurtosis analysis, indicated a non-normal distribution of the MTL data. Model validation showed that fifth-degree polynomial regression provided a robust fit with high fitted and predicted R^2^ values, indicating strong predictive power. Hill-climbing optimization was used to fine-tune the welding parameters, identifying an optimal spindle speed of 1100 rpm and a welding speed of 332 mm/min, which was predicted to achieve an MTL of 16,852 N. Response surface analysis confirmed the effectiveness of the identified parameters and demonstrated their significant influence on the MTL. These results suggest that the applied polynomial regression model and optimization approach are effective tools for improving the performance and reliability of the FSW process.

## 1. Introduction

Friction Stir Welding (FSW), introduced in the early 1990s by Thomas et al. [1], is a solid-state joining technique that has gained widespread attention for its ability to produce high-quality joints in lightweight and difficult-to-weld alloys, notably aluminum [2]. Operating below the melting temperature of the base material, FSW reduces common defects found in fusion welding—such as porosity, hot cracking, and distortion—resulting in improved mechanical properties and structural integrity [3]. Due to these advantages, it has found extensive applications in transportation, aerospace, and marine industries [4,5,6]. However, determining the optimal set of parameters—rotational speed, traverse speed, axial force, tool geometry, and cooling strategy—remains challenging, as these factors must be balanced to achieve the desired mechanical strength, microstructural refinement, and minimal defect formation [7,8,9].

Early optimization strategies often relied on trial-and-error approaches combined with classical statistical tools (e.g., Taguchi, ANOVA), which provided some guidance but were time-consuming and limited in handling the nonlinear and high-dimensional parameter interactions of FSW [10,11,12]. For instance, Sabry et al. [7] and Kesharwani et al. [8] employed statistical methodologies to identify welding conditions that improved tensile properties and reduced defects. While these approaches could pinpoint useful parameter trends, they typically demanded substantial experimental efforts. Similarly, preliminary models or linear approximations struggled with complex parameter spaces, especially when multiple performance criteria had to be considered simultaneously [13,14,15].

As computational resources improved, finite element modeling (FEM) and numerical simulations contributed insights into temperature fields, material flow, and residual stresses. He et al. [3] and Jasim et al. [16], for example, integrated FEM with data-driven methods to propose parameter sets that enhanced joint performance. Although simulations helped reduce the trial-and-error burden, FEM models are often computationally expensive and may require extensive calibration, limiting their scalability and adaptation to new welding conditions [17,18,19,20,21].

The advent of artificial intelligence (AI) and machine learning (ML) techniques provided new avenues for more accurate and cost-effective process optimization [6,13,22]. By leveraging historical or experimentally obtained datasets, ML models can capture complex, nonlinear parameter-response relationships. Myśliwiec et al. [22] applied Random Forest, XGBoost, and MLP models to optimize the FSW of 2024-T3 aluminum alloy, demonstrating that advanced ML algorithms could accurately predict mechanical outputs and suggest improved parameter combinations. Similarly, Cho et al. [13] and Albaijan et al. [14] showed that artificial neural network-based predictions not only improved accuracy but also reduced guesswork in selecting parameter sets that enhanced tensile strength and joint reliability.

Nonetheless, ML approaches often rely on extensive, high-quality datasets and can pose interpretability challenges. Vidakis et al. [23] found that integrating data-driven models with domain knowledge (e.g., polynomial regression or RSM-based insights) reduced the need for large-scale experimentation. Mishra et al. [19] and Sambath et al. [24] highlighted that while ML excels at identifying trends and relationships, its predictive power is sensitive to data diversity and preprocessing quality. Chadha et al. [25] successfully implemented ML-based defect prediction yet noted that specialized datasets and sensor feedback were needed for real-time adaptation, adding complexity to practical implementation.

Metaheuristic and evolutionary algorithms have shown promise in exploring large parameter spaces and identifying global optima [26,27,28]. These methods can balance multiple performance criteria—such as tensile strength, microstructural uniformity, and energy efficiency—more flexibly than classical optimization techniques. Prabhakar et al. [29] and Kubit et al. [30] employed multi-objective optimization frameworks to improve joint efficiency and processing time, outperforming baseline conditions derived from simpler approaches. Rana et al. [26] reported that while hybrid evolutionary algorithms could identify global optima, the computational overhead and complexity in tuning these algorithms were not trivial.

To mitigate these challenges, researchers have explored hybrid strategies combining polynomial regression, RSM, ML, and metaheuristics to achieve interpretability, data efficiency, and accuracy. Yaknesh et al. [31] and Rao et al. [32] demonstrated that polynomial models, when integrated with evolutionary searches, provided robust optimization results without the heavy computational demands typical of complex ML models. Sabry et al. [15] and Babalola et al. [33] found that using polynomial fitting as a preliminary modeling step not only improved interpretability but also served as a strong foundation for subsequent ML-driven refinement.

Bayesian optimization, transfer learning, and multi-criteria decision-making frameworks further enhance adaptability, enabling parameter identification that can transfer across materials or joint configurations with minimal additional experimentation [6,34,35,36,37]. Sengupta et al. [6], for example, demonstrated how transfer learning could reuse knowledge from one alloy system to expedite optimization in another. Kumar et al. [11] used multi-criteria decision-making to incorporate human expertise and manufacturing constraints directly into the decision-making process, offering a more holistic perspective.

Despite these advancements, key gaps remain. Many studies reported substantial improvements in specific metrics—such as achieving a 10–20% increase in tensile strength or reduced defects [13,22,38]—but often under constrained conditions or tailored datasets. Scalability, generalizability, and adaptability to limited data scenarios remain pressing concerns. Likewise, sophisticated ML or metaheuristic approaches might require careful tuning and might not always be justifiable for certain industrial environments [36,39,40]. Ensuring that enhancements in one property (e.g., tensile strength) do not compromise others (e.g., ductility or fatigue life) is another ongoing challenge [29,40].

This study proposes a hybrid approach that combines polynomial regression modeling with a hill-climbing optimization technique to predict and enhance the maximum tensile load (MTL) of FSW joints. Polynomial regression can capture higher-order interactions while maintaining interpretability and reducing complexity, and hill-climbing provides a straightforward iterative procedure for refining parameters and approaching near-optimal solutions. Unlike pure ML models that require extensive data or metaheuristic methods that may be computationally demanding, this strategy aims for a balanced solution. By leveraging polynomial modeling as a starting point and then applying hill-climbing optimization, the approach reduces both experimental and computational burdens while delivering strong predictive capabilities. Thus, it can potentially match or exceed the improvements reported in the literature while offering greater transparency, adaptability, and reduced cost—key factors in advancing robust FSW parameter optimization.

## 2. Materials and Methods

**Figure 1:**
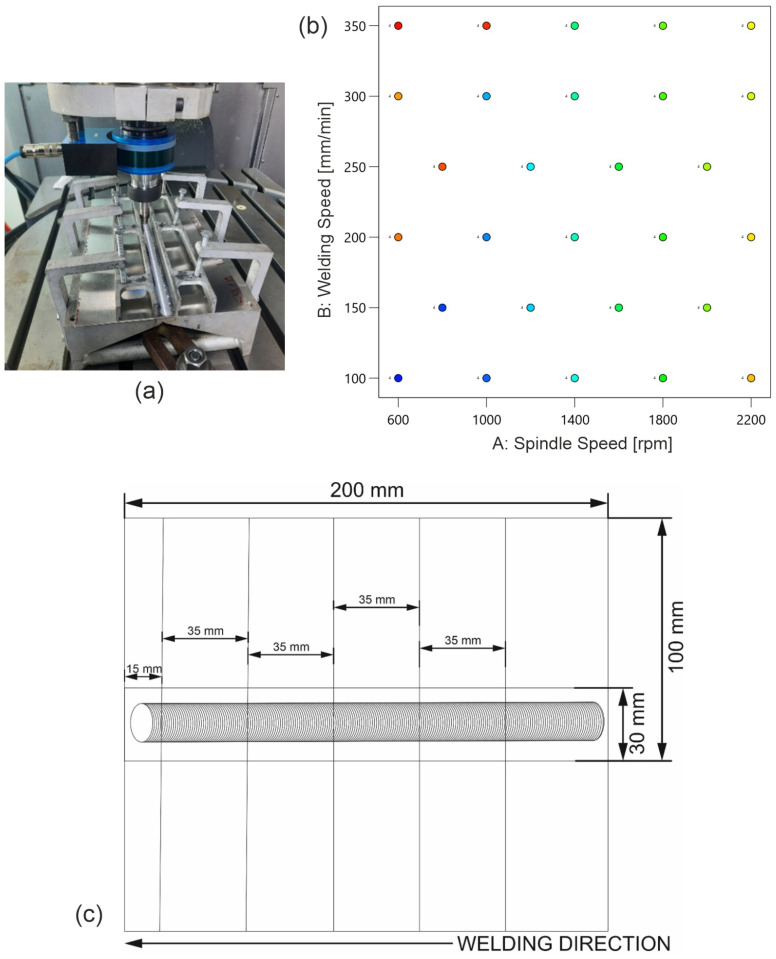
(**a**) FSW machine, (**b**) factor plan of the FSW process, and (**c**) configuration of the welded panels (unit mm).

The friction stir welding process was conducted using AA2024-T3 aluminum sheets with a thickness of 1.5 mm. The experiments were performed on a Makino PS95 CNC milling machine (Figure 1a) using a commercially available tool with the geometric parameters shown in Table 1. The plates, which were 200 mm long and 100 mm wide, were joined along the rolling line. The joints were configured in a lap joint arrangement with a lap width of 30 mm. A factorial design was used to plan the experiment. The design matrix, shown in Figure 1b, included 28 experimental points with varying spindle speeds from 600 to 2200 rpm and welding speeds from 100 to 350 mm/min. The technological parameters for each test run, along with the measured strength of the FSW joints. The selected range of spindle and welding speeds was determined through a comprehensive literature review and preliminary experimental trials. Prior research on AA2024-T3 and analogous aluminum alloys indicated that these ranges are optimal for attaining defect-free welds with high mechanical performance. Furthermore, in-house trials were conducted to refine the process window, thereby ensuring that the selected parameters encompassed the regions where the maximum tensile strength of the joints could be obtained. This factorial design allowed a comprehensive analysis of the effects of spindle and welding speeds on the tensile strength of the FSW joints, ensuring a robust assessment of the optimum welding conditions. Each joint was then divided into four sections for tensile testing. The specimens were precisely cut using a wire electrical discharge machining (EDM) technique to minimize the influence of external forces on the joint structure. The cutting process followed the configuration shown in Figure 1c. Each of the four specimens was mounted on a ZWICK/ROELL Z100 universal testing machine to evaluate the bond strength. The experimental results were analyzed using Design Expert 12 software, and a fifth-degree polynomial regression model was developed to predict and optimize the technological parameters in the FSW process.

### Evaluation of the Experimental Model

The FSW process was implemented for a range of parameters: tool speed from 600 to 2200 rpm and welding speed from 100 to 350 mm/min. The independent variables were coded and presented in Table 2. The dependent variable was the response or strength of the FSW lap joint. The resulting load capacities ranged from 1912 to 15,336 N (Table 3).

**Figure 2:**
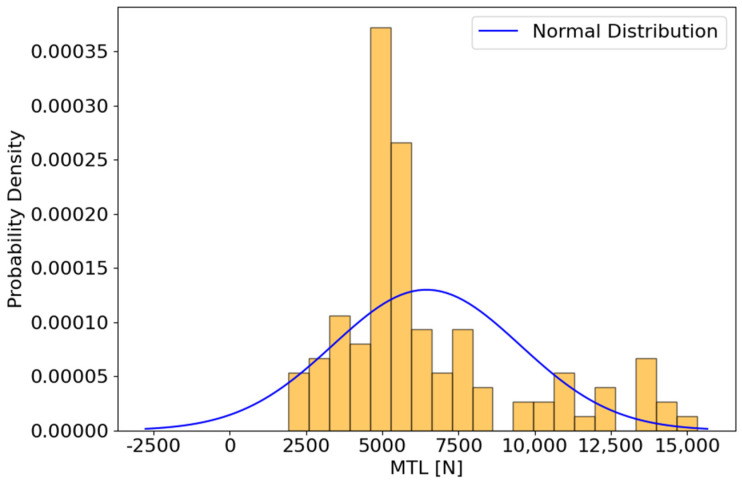
Histogram of maximum tensile load (MTL) results from the FSW process. The analysis indicates a right-skewed distribution, showing that the data are not normally distributed.

The histogram of the ultimate maximum tensile load, hereafter referred to as MTL, measurements (Figure 2) from the FSW process were analyzed along with statistical tests to assess the normality of the data distribution. The histogram shows a right-skewed distribution of MTL values, with the majority of measurements concentrated between 2500 N and 7500 N. The peak frequency occurs around 5000 N, and there is a noticeable decrease in frequency towards higher MTL values. To statistically evaluate the normality of the data, the Shapiro–Wilk test was performed. The test statistic is 0.861 with a *p*-value of 7.62 × 10^−9^, indicating that the MTL data do not follow a normal distribution, and the null hypothesis (H0) is rejected. This result is visually confirmed by superimposing a normal distribution curve on the histogram, which highlights significant departures from normality. Further analysis of the kurtosis of the data yields a value of 0.764. This positive kurtosis indicates that the distribution has heavier tails and a sharper peak compared to a normal distribution, contributing to the observed skewness. In addition, the histogram shows several outliers at higher MTL values, especially above 10,000 N, further supporting the non-normality of the data.

**Figure 3:**
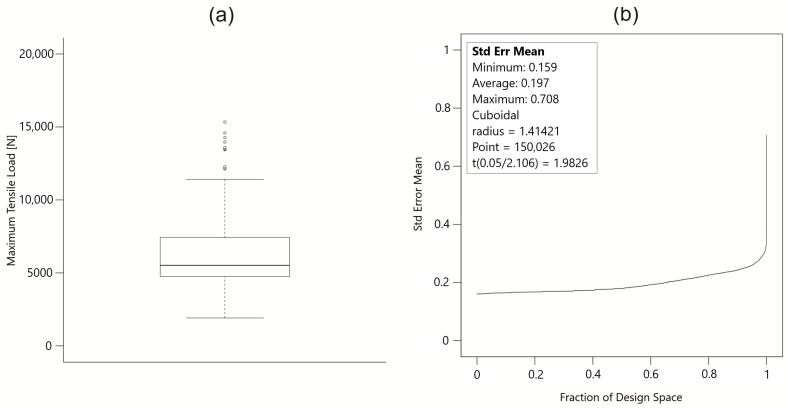
(**a**) Box plot of maximum tensile load (MTL) results from the FSW process. (**b**) Fraction of design space (FDS) graph for standard error mean.

The box plot of the maximum tensile load (MTL) measurements (Figure 3a) from the FSW process was analyzed to identify potential outliers and assess the overall data distribution. The box plot shows that the majority of the MTL values are concentrated within the interquartile range (IQR), with the median value in the lower half of the IQR, indicating a slight skew in the data distribution. The box plot analysis highlights several high MTL values that are considered outliers. However, these outliers are critical to understanding the conditions that lead to exceptionally high tensile strengths in welds. Rather than dismissing these values, further investigation is warranted to explore the specific parameters and conditions that resulted in these superior weld strengths. This information could be critical to optimizing the FSW process and achieving more reliable and higher-quality welds. The final step in evaluating the quality of the experimental model was to plot the FDS. The graph (Figure 3b) plots the standard error mean (Std Error Mean) as a function of the fraction of the design space (FDS). This type of graph is typically used to evaluate the quality of an experimental design [41]. The X-axis represents the fraction of the design space, while the y-axis represents the standard error of the mean. The design space is defined as a cube, indicating a rectangular area of variable space. The radius of 1.41421 indicates that the space is constructed considering the Euclidean distance. The analysis was performed on a very large number of points (150,026), which increases the accuracy of the evaluation. The t-Student value indicates the critical value for the t-distribution at a given confidence level, which is used to assess statistical significance. The design is well constructed and accurate in the central regions of the design space, indicating good model quality in these regions. The increase in standard error at the edges is typical and indicates potentially lower model reliability in these regions but requires attention at the edges of the design space.

## 3. Creating a Polynomial Regression Model

### 3.1. Principles of Polynomial Regression

Polynomial regression is an extension of linear regression that is used to model the relationship between a dependent variable *y* and one or more independent variables *x* by fitting a polynomial equation to the observed data. This type of regression is particularly useful when the relationship between the variables is nonlinear. The polynomial regression model of degree *n* can be written as:

(1) $$y=\beta_{0}+\beta_{1}x+\beta_{2}x^{2}+\beta_{3}x^{3}+\cdots +\beta_{n}x^{n}+\epsilon$$

where *y* is the dependent variable, *x* is the independent variable, *β*_0_, *β*_1_, *β*_2_, …, *β_n_* are the coefficients of the polynomial to be estimated from the data, and *ϵ* is the error term representing the difference between the observed and predicted values [42]. The polynomial regression process involves several steps. First, data points (*x_i_*, *y_i_*) are collected for *i* = 1, 2, …, *m*, where *m* is the number of observations. Next, the degree *n* of the polynomial is chosen, with a higher degree polynomial fitting the data better but potentially leading to overfitting. A design matrix *X* is then constructed that contains the powers of the independent variable *x*. For a polynomial of degree *n*, the design matrix is *X*:

(2) $$X=\left[\begin{array}{ccccc} 1 & x_{1} & x_{1}^{2} & \ldots & x_{1}^{n}\\ 1 & x_{2} & x_{2}^{2} & \ldots & x_{2}^{n}\\ \vdots & \vdots & \vdots & \ddots & \vdots \\ 1 & x_{m} & x_{m}^{2} & \ldots & x_{m}^{n} \end{array}\right]$$

The coefficients *β* are estimated using the least squares method, which minimizes the sum of the squared differences between the observed values $y_{i}$ and the values predicted by the polynomial. The coefficients are given by:

(3) $$\beta ={\left(X^{T}X\right)}^{-1}X^{T}y$$

where $X^{T}$ is the transpose of the design matrix *X*, and *y* is the vector of observed values. Once the coefficients are estimated, the polynomial equation can be used to predict the values of the dependent variable for new values of the independent variable *x* [43,44,45]. To illustrate the process, consider fitting a fifth-degree polynomial regression model to a set of data points (*x*_1_, *y*_1_), (*x*_2_, *y*_2_), …, (*x*_*m*_, *y*_*m*_). The fifth-degree polynomial regression model can be written as:

(4) $$y=\beta_{0}+\beta_{1}x+\beta_{2}x^{2}+\beta_{3}x^{3}+\beta_{4}x^{4}+\beta_{5}x^{5}+\epsilon$$

The data points (*x_i_*, *y_i_*) are collected for *i* = 1, 2, …, *m*. The design matrix *X* is formulated to include the powers of the independent variable *x*. For a fifth-degree polynomial, the design matrix *X* is constructed as follows:

(5) $$X=\left[\begin{array}{cccccc} 1 & x_{1} & x_{1}^{2} & x_{1}^{3} & x_{1}^{4} & x_{1}^{5}\\ 1 & x_{2} & x_{2}^{2} & x_{2}^{3} & x_{2}^{4} & x_{2}^{5}\\ \vdots & \vdots & \vdots & \vdots & \vdots & \vdots \\ 1 & x_{m} & x_{m}^{2} & x_{m}^{3} & x_{m}^{4} & x_{m}^{5} \end{array}\right]$$

Once the coefficients are estimated, the polynomial equation is used to predict the values of the dependent variable for new values of the independent variable *x*:

(6) $$\hat{y}=\beta_{0}+\beta_{1}x+\beta_{2}x^{2}+\beta_{3}x^{3}+\beta_{4}x^{4}+\beta_{5}x^{5}+\epsilon$$

The Fit Summary Table 4 compares different polynomial regression models for the MTL data from the FSW process. Sequential *p*-values indicate that cubic, quartic, fifth, and sixth polynomial terms significantly improve the model fit (*p* < 0.0001), meaning that the addition of these terms provides a statistically significant improvement to the model. The lack of fit *p*-value is less than 0.0001 for all models, indicating a statistically significant lack of fit. This indicates that the models do not perfectly capture all the underlying patterns in the data. However, it is common in complex real-world data for models to show some lack of fit. The adjusted R^2^, which accounts for model complexity, increases from 0.3877 for the linear model to 0.9752 for the sixth-degree polynomial. Similarly, the predicted R^2^, which indicates the predictive power of the model, also increases with model complexity. The fifth-degree polynomial model is recommended with an adjusted R^2^ of 0.9489 and a predicted R^2^ of 0.9390, providing an excellent balance between model fit and predictive accuracy. Although the sixth-degree polynomial has slightly higher R^2^ values, it is noted as aliased, suggesting potential overfitting or multicollinearity issues. In conclusion, while the lack of fit is statistically significant for all models, the fifth-degree polynomial model stands out with high fitted and predicted R^2^ values, indicating strong model performance and predictive power. This makes it the optimal choice for accurately modeling and predicting MTL data in the FSW process.

Table 5 presents key indicators of the quality of the polynomial regression model used to optimize the FSW process. These indicators include the standard error, VIF, R^2^, and power of the model for each term. The standard error measures the variability of the regression coefficient estimates. In a balanced design, the standard errors for different terms should be similar. In this case, the standard errors for all terms are relatively small, indicating robust estimates in the model. The lowest standard error is 0.1357 for term B (welding speed), and the highest is 0.2453 for term A^2^ (spindle speed squared). The Variance Inflation Factor (VIF) measures the degree of collinearity between independent variables. The ideal VIF value is 1.0, with values greater than 10 indicating significant collinearity, which could lead to problems with coefficient estimation. All terms have VIF values close to 1.0, indicating no significant collinearity in the model. R^2^ measures the fit of the model and indicates the proportion of variance in the dependent variable that is explained by the independent variables. For an ideal polynomial regression model, the R^2^ values for individual terms should be close to 0 to avoid overfitting. The R^2^ values for all terms are very low, indicating no overfitting and suggesting that the model fits well without unnecessary complexity. The power of the statistical test indicates the ability to detect a true effect if it exists. High power (close to 100%) indicates that the model is highly effective in detecting the influence of process variables on joint strength. All terms have a power level of 99.9%, indicating that the model is very effective in detecting the effects of the process variables. The quality assessment of the polynomial regression model for the FSW process indicates that the model is well fitted and does not have problems with collinearity or overfitting. The low standard errors and VIF values, along with the high power values, demonstrate the robustness and effectiveness of the model. The R^2^ values suggest that the model is well calibrated without unnecessary complexity, which is beneficial for the interpretation of results and practical application.

### 3.2. ANOVA for Fifth Model

An analysis of variance was performed on the accepted fifth-degree polynomial regression model. The ANOVA Table 6 for the 5th degree polynomial model of the MTL data from the FSW process provides a comprehensive analysis of the sources of variation and their statistical significance. The overall model is highly significant, with an F-value of 104.12 and a *p*-value of less than 0.0001, indicating that the model effectively explains a significant portion of the variability in MTL. Several terms in the model are identified as statistically significant with *p*-values less than 0.05. These include the main effect of spindle speed (A) and numerous higher-order interactions and polynomial terms such as AB (spindle speed * welding speed), A^2^, B^2^, A^3^, B^3^, A^4^, B^4^, A^5^, and B^5^. The significance of these terms suggests that both the main effects and complex interactions between spindle speed and welding speed are critical in determining MTL. However, some terms, such as the main effect of welding speed (B) and interactions such as A^2^B, A^3^B, A^2^B^3^, and A^4^B, are not statistically significant, as indicated by their *p*-values greater than 0.05. These non-significant terms do not contribute meaningfully to the model, suggesting that they could be excluded in future model refinement to improve simplicity without sacrificing predictive power. The F-value for lack of fit is 49.80, with a *p*-value of less than 0.0001, indicating a significant lack of fit. This suggests that the model does not perfectly capture all the underlying variability in the data and that there may be other factors or interactions influencing MTL that are not included in the model.

The fit statistics for the fifth-degree polynomial model (Table 7) applied to the MTL data from the FSW process indicate strong model performance. The model achieves a high R^2^ value of 0.9581, explaining 95.81% of the variability in MTL. The adjusted R^2^ of 0.9489 and the predicted R^2^ of 0.9390 are in close agreement, indicating excellent predictive accuracy and minimal overfitting. The standard deviation of the residuals is 694.15, and the coefficient of variation is 10.76%, indicating low variability relative to the MTL mean of 6451.97. In addition, the adequate precision ratio of 40.7155 far exceeds the desirable threshold of 4, confirming a strong signal-to-noise ratio.

The final regression equation (Table 8) for predicting MTL in the FSW process is expressed in terms of the actual factors, specifically spindle speed and welding speed. This equation allows accurate predictions by substituting the specified values of these factors. It is important to note that the coefficients are scaled to the units of each factor, and the intercept is not centered in the design space.

## 4. Results

The applied regression model was used to predict maximum tensile load, hereafter referred to as MTL values. In addition, a series of diagnostic tests were performed on the applied model. The experimental results, along with the predicted MTL values from the fifth-degree polynomial regression model and associated metrics, are presented in Table 9. The table lists the actual MTL values from the experiments, the predicted maximum tensile load values from the regression model, the residuals (differences between actual and predicted values), leverage values indicating the influence of each data point on the model, internally and externally studentized residuals for detecting outliers, Cook’s Distance for identifying influential observations, and DFFITS values for assessing the influence of each observation on the fitted values.

### 4.1. Model Diagnostics

**Figure 4:**
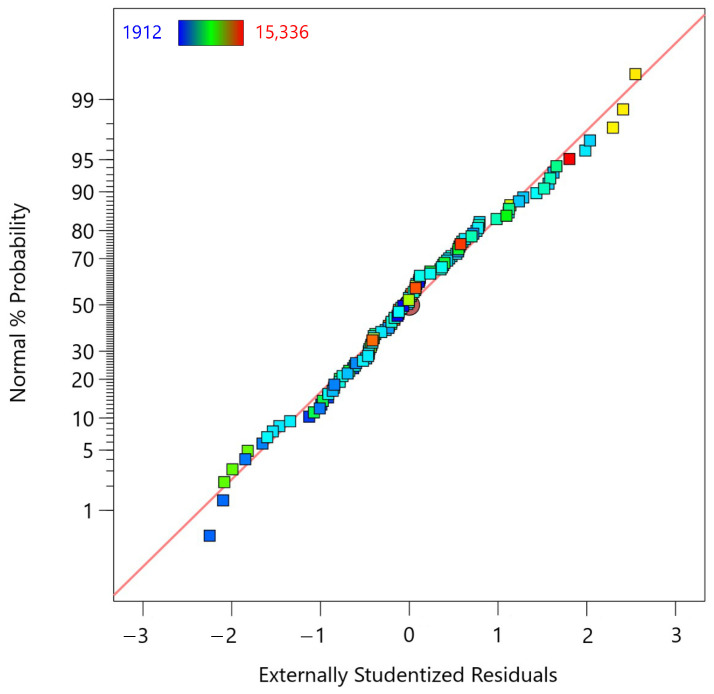
Normal probability plot of residuals for the fifth-degree polynomial regression model applied to MTL data.

For the accepted regression model, diagnostic tests were performed to assess its validity. The first diagnostic test is the Normal Probability Plot of Residuals (Figure 4), which assesses whether the residuals from the fifth-degree polynomial regression model for the MTL data are normally distributed. In this plot, the externally studentized residuals are plotted on the *x*-axis, while the corresponding normal cumulative probabilities are plotted on the *y*-axis. Most of the residuals are close to the red reference line, indicating that they follow a normal distribution. However, some deviations from normality are observed, especially in the tails of the distribution. Some residuals, especially those on the far right, deviate significantly from the line, indicating potential outliers or deviations from normality.

**Figure 5:**
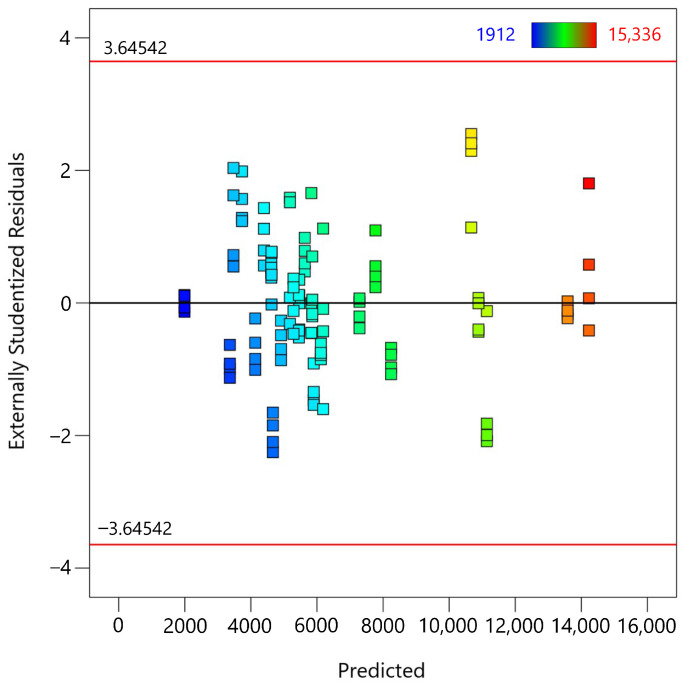
Residuals vs. predicted values plot for the fifth-degree polynomial regression model applied to MTL data.

The second diagnostic test is the residuals versus predicted values plot (Figure 5), which evaluates whether the residuals are randomly distributed, indicating a good fit for the regression model. The plot shows the externally studentized residuals on the y-axis and the predicted MTL values on the x-axis. Most of the residuals are randomly scattered around the horizontal line at zero, indicating that the model captures the data well with no obvious patterns. The absence of a clear pattern or trend in the residuals indicates that there is no significant nonlinearity or heteroscedasticity (nonconstant variance). This random scatter supports the assumption that the residuals are independent and identically distributed. The plot includes red lines at ±3.65 standard deviations. Note that none of the residuals exceed these thresholds, indicating that there are no extreme outliers. The concentration of the residuals around the zero line, with no discernible patterns, confirms that the model is a good fit for most of the data points.

**Figure 6:**
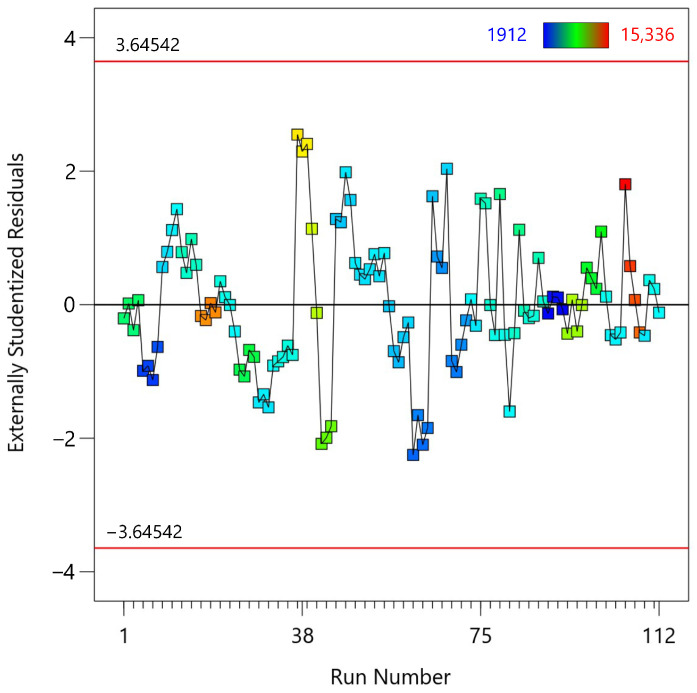
Residuals vs. run order plot for the fifth-degree polynomial regression model applied to MTL data.

The third diagnostic test is the residuals versus run order plot (Figure 6), which assesses whether the residuals are randomly distributed across the sequence of observations, indicating the absence of temporal or sequence bias in the model. The plot plots the externally studentized residuals on the y-axis and the run number on the *x*-axis. Most of the residuals are scattered around the horizontal line at zero, indicating that there is no clear pattern or trend over the sequence of observations. Although there is some variation in the residuals, there is no consistent pattern or trend that would suggest systematic errors related to run order. This lack of a discernible trend suggests that the residuals are independent of run order, supporting the assumption that there is no autocorrelation in the residuals. The Durbin-Watson statistic, which tests for the presence of autocorrelation in the residuals, is 0.8776 with an autocorrelation value of 0.5609. A Durbin-Watson value close to 2 indicates no autocorrelation, while values significantly lower or higher indicate positive or negative autocorrelation, respectively. The observed value (0.8776) indicates some positive autocorrelation, which may require further investigation. The plot includes red lines at ±3.64 standard deviations, and none of the residuals exceed these thresholds, indicating that there are no extreme outliers.

**Figure 7:**
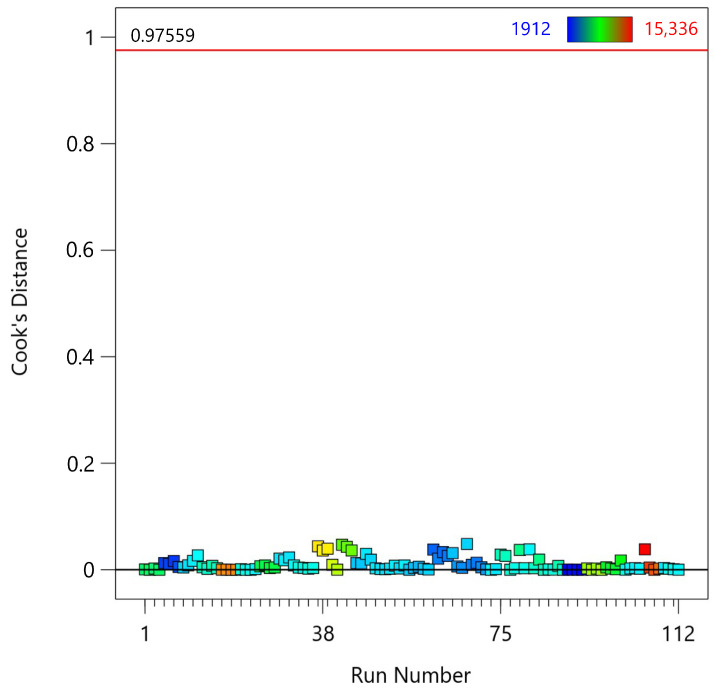
Cook’s Distance plot for the fifth-degree polynomial regression model applied to MTL data.

The fourth diagnostic test is the Cook distance plot (Figure 7), which evaluates the influence of each observation on the regression model. Cook’s Distance measures how much the regression coefficients change when a particular observation is removed, helping to identify influential data points that may disproportionately affect the model [46]. Cook’s Distance for the *i*-th observation is calculated using the following formula:

(7) $$D_{i}=\frac{e_{i}^{2}}{p\cdot MSE}\left(\frac{h_{i}}{{\left(1-h_{i}\right)}^{2}}\right)$$

where $D_{i}$ is the Cook’s Distance for the *i*-th observation. $e_{i}$ is the residual for the *i*-th observation (i.e., the difference between the observed and fitted values for that observation). *p* is the number of parameters in the model, including the intercept. *MSE* is the mean squared error of the regression model. $h_{i}$ is the leverage of the *i*-th observation, which is the *i*-th diagonal element of the hat matrix *H* = *X*(*X*′*X*) − 1*X*′. Most observations have Cook’s Distance values close to zero, indicating that they have minimal influence on the regression model. The red line at Cook’s Distance of 0.976 serves as a threshold; points above this line are considered highly influential. In this plot, none of the observations exceed the Cook’s Distance threshold of 0.976, indicating that there are no highly influential data points in the data set.

**Figure 8:**
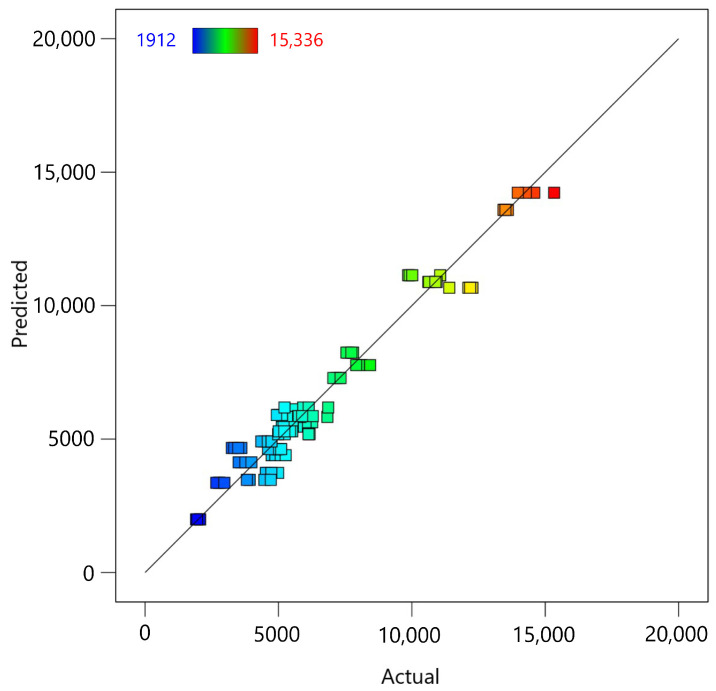
Predicted vs. actual values plot for the fifth-degree polynomial regression model applied to MTL data.

The fifth diagnostic test is the predicted vs. actual plot (Figure 8), which evaluates how well the regression model predicts the observed data. The distribution of points along the line indicates a strong correlation between predicted and actual values, confirming the model’s ability to accurately predict MTL.

**Figure 9:**
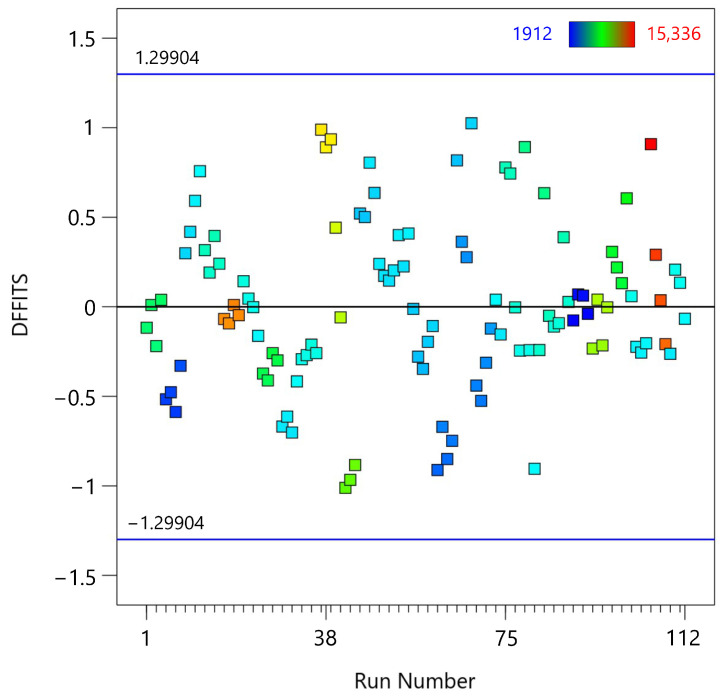
DFFITS vs. run number plot for the fifth-degree polynomial regression model applied to MTL data.

The sixth diagnostic test is the DFFITS vs. Run Number plot (Figure 9), which examines the influence of each observation on the fitted values of the regression model. DFFITS (Difference in Fits) measures how much an observation affects the fitted value and is used to identify influential points that may disproportionately affect the model. DFFITS is a diagnostic measure that quantifies the influence of a single observation on the fitted values of the regression model. It calculates the change in the predicted value when an observation is excluded from the model. The formula for DFFITS is given by:

(8) $${DFFITS}_{i}=\frac{{\hat{y}}_{i}-{\hat{y}}_{i(-i)}}{s_{\left(-i\right)}\sqrt{h_{i}}}$$

where ${\hat{y}}_{i}$ is the predicted value with all observations included. ${\hat{y}}_{i(-i)}$ is the predicted value with the *i*-th observation excluded. $s_{\left(-i\right)}$ is the standard error of the regression with the *i*-th observation excluded. $h_{i}$ is the leverage of the *i*-th observation. Many of the DFFITS values lie around zero, indicating that individual observations generally have a minimal influence on the fitted values of the model. As observations move further from the zero line, they have a greater impact on the model’s fitted values. The horizontal blue lines at ±1.29904 serve as thresholds, calculated based on the formula $\pm 2\sqrt{\frac{p}{n}}$, where *p* is the number of predictors (including the intercept), and *n* is the number of observations. Values beyond these lines suggest influential observations [47]. The plot shows that none of the observations exceed these thresholds, indicating that there are no highly influential data points in the data set. This suggests that the model is robust and not unduly influenced by any single observation.

**Figure 10:**
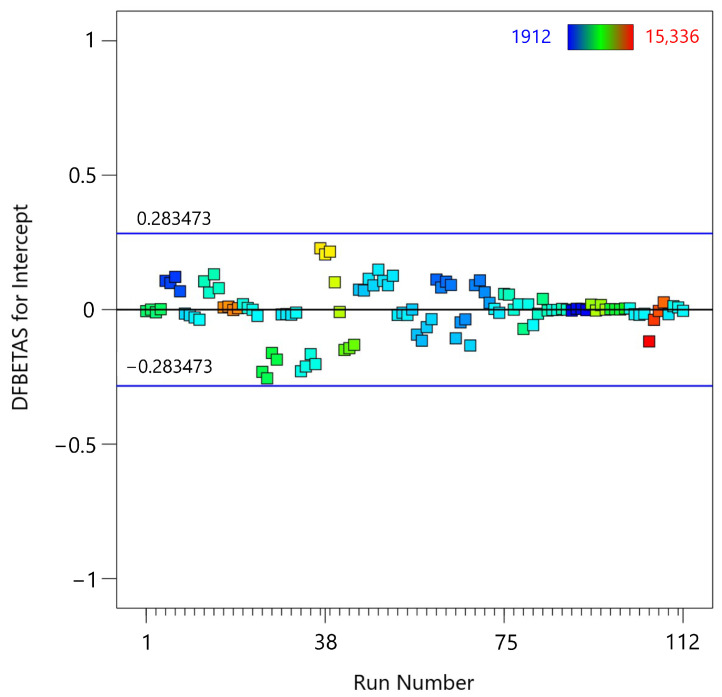
DFBETAS vs. run number plot for the fifth-degree polynomial regression model applied to MTL data.

The next diagnostic test is the DFBETAS vs. Run Number plot (Figure 10), which examines the influence of each observation on the estimated regression coefficients. DFBETAS, which stands for “Difference in Beta”, is a diagnostic measure that assesses the impact of each individual data point on the estimated regression coefficients. Specifically, it measures the change in a regression coefficient when an observation is omitted from the analysis. The formula for DFBETAS is:

(9) $${DFBETAS}_{ij}=\frac{{\hat{\beta }}_{j}-{\hat{\beta }}_{j(-i)}}{s_{\left(-i\right)}\sqrt{{\left(X\prime X\right)}_{jj}^{-1}}}$$

where ${\hat{\beta }}_{j}$ is the estimated coefficient for predictor *j* with all observations included. ${\hat{\beta }}_{j(-i)}$ is the estimated coefficient for predictor *jj* with the *i*-th observation excluded. $s_{\left(-i\right)}$ is the standard error of the regression with the *i*-th observation excluded. ${\left(X\prime X\right)}_{jj}^{-1}$ is the *j*-th diagonal element of the inverse of the design matrix *X*′*X*.

DFBETAS values indicate how much an individual observation influences the regression coefficients. Large DFBETAS values indicate that the observation has a significant impact on the corresponding coefficient, potentially indicating an influential data point that may disproportionately affect the model’s estimates. In the plot, the DFBETAS values for the intercept are plotted on the *y*-axis, and the run number is plotted on the *x*-axis. Many of the DFBETAS values are around zero, indicating that most observations have little to no effect on the estimated regression coefficients. The horizontal blue lines at ±0.284573 serve as thresholds, calculated using the formula $\pm 2/\sqrt{n}$ where *n* is the number of observations. Values beyond these lines indicate that the corresponding observations have a significant impact on the model’s coefficients [48]. The plot shows that all observations fall within the thresholds of ±0.284573, indicating that none of the data points have an undue influence on the regression coefficients. This indicates that the model is stable, and the coefficients are not overly sensitive to any single observation. 

Both measures are useful for identifying influential data points, but they provide different perspectives on how an observation affects the regression model. DFFITS is concerned with overall prediction accuracy, while DFBETAS is concerned with the stability of the regression coefficients.

### 4.2. Principles of Hill-Climbing Algorithm

Hill climbing is an optimization algorithm used to find the best solution to a problem by iteratively improving the current solution based on a fitness function. Starting with an arbitrary initial solution, the algorithm evaluates its fitness and generates neighboring solutions by making small changes. It then selects the neighbor with the best fitness as the new current solution. This process is repeated until a stopping criterion is met, such as a fixed number of iterations, a time limit, or when no better neighbors are found. Hill climbing is a local search algorithm that focuses on improving the current solution and takes a greedy approach by always moving to the best neighbor solution. However, it can become stuck in local optima, i.e., solutions that are better than their neighbors but not the best overall. Variants such as Steepest Ascent, Stochastic, and First-Choice Hill Climbing help to solve this problem. Hill climbing is easy to implement and efficient for small problem spaces, making it useful in applications such as artificial intelligence, operations research, and machine learning.

The process begins with an initial guess

(10) $$S_{0}=initial\;guess.$$

The fitness of a solution *S* is evaluated using a fitness function *f*(*S*), which maps the solution to a real number indicating its quality.

f:S→R.

The algorithm generates a set of neighboring solutions *N*(*S*) by making small perturbations to the current solution *S*.

(11) $$N\left(S\right)=\{S^{\prime }|S^{\prime }is\;a\;neighbor\;of\;S\}$$

Among the generated neighbors, the algorithm selects the neighbor *S′* with the optimal fitness value, either the highest for maximization problems or the lowest for minimization problems.

(12) $$S^{\prime }={argmax}_{S^{\prime }\in N\left(S\right)}f(S^{\prime })\;(\mathrm{for}\;\mathrm{maximization})\;S^{\prime }={argmin}_{S^{\prime }\in N\left(S\right)}f(S^{\prime })\;(\mathrm{for}\;\mathrm{minimization})$$

If the best neighbor *S*′ improves the fitness function compared to the current solution *S*, the algorithm updates *S* to *S*′.

(13) $$\mathrm{If}\;f\left(S^{\prime }\right)>f(S)\;(\mathrm{for}\;\mathrm{maximization}),\;\mathrm{then}S=S^{\prime }\;\mathrm{If}\;f\left(S^{\prime }\right)<f(S)\;(\mathrm{for}\;\mathrm{minimization}),\;\mathrm{then}S=S^{\prime }$$

The algorithm iterates through the steps of generating neighbors and selecting the best one until a predefined stopping criterion *T* is met. This criterion could be a fixed number of iterations, a time limit, or the absence of further improvements [49,50].

## 5. Optimization

**Figure 11:**
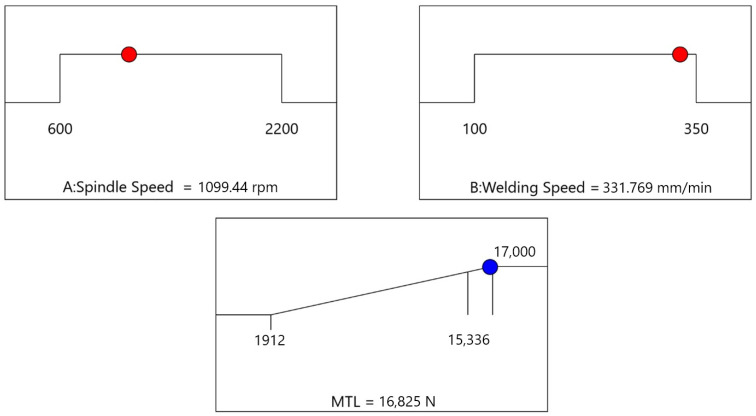
Optimization results for welding parameters using hill climbing.

For our model, hill-climbing optimization was performed to fine-tune the welding parameters to achieve the best possible MTL. The process involved starting with an initial set of welding parameters and iteratively adjusting these parameters to explore their neighboring values. At each step, the fitness function, defined as the MTL, was evaluated to identify the optimal combination of welding parameters. By selecting the parameters that maximized the MTL, the algorithm iteratively moved toward the best solution, thereby improving the overall performance and reliability of the welding process. This approach ensured that the parameters were effectively optimized, resulting in the highest achievable MTL for the given welding conditions. The optimal parameters identified through this process are a spindle speed of 1100 rpm and a welding speed of 332 mm/min, which are predicted to achieve an MTL of 16,852 N. Figure 11 shows the optimization parameters.

**Figure 12:**
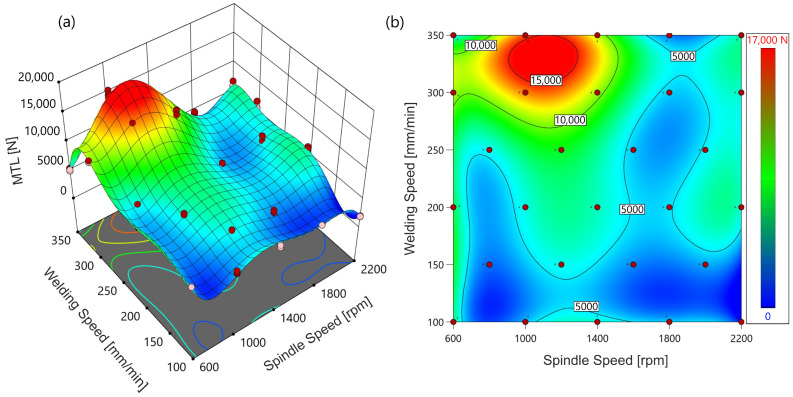
Response surface plot (**a**) and contour plot (**b**) for MTL as a function of spindle speed and welding speed. Different colors indicate MTL values.

The response surface plot (Figure 12) shows the relationship between MTL and two key welding parameters: spindle speed (rpm) and welding speed (mm/min). 

The surface plot has regions of different heights that indicate how changes in spindle speed and welding speed affect the MTL. The highest region of the surface corresponds to the maximum MTL values, represented by the red and yellow areas on the plot. The plot shows a peak MTL value around the spindle speed of approximately 1100 rpm and welding speed of approximately 332 mm/min, which is consistent with the optimal parameters determined by the hill-climbing optimization.

## 6. Confirmation Test

The specimen subjected to the confirmation test was FSW in a lap joint configuration using the optimal parameters identified by the optimization process: a spindle speed of 1100 rpm and a welding speed of 332 mm/min. The test procedure followed the same protocol as described in the Materials and Methods section to ensure consistency in the evaluation of the welds. The measured load capacity for the FSW sample in the confirmation test ranged from 16,200 to 17,300 N, which is in good agreement with the predicted values from the optimization model. This confirms the accuracy and reliability of the optimization process for these specific parameters.

### 6.1. Macro and Microstructure Analysis

**Figure 13:**
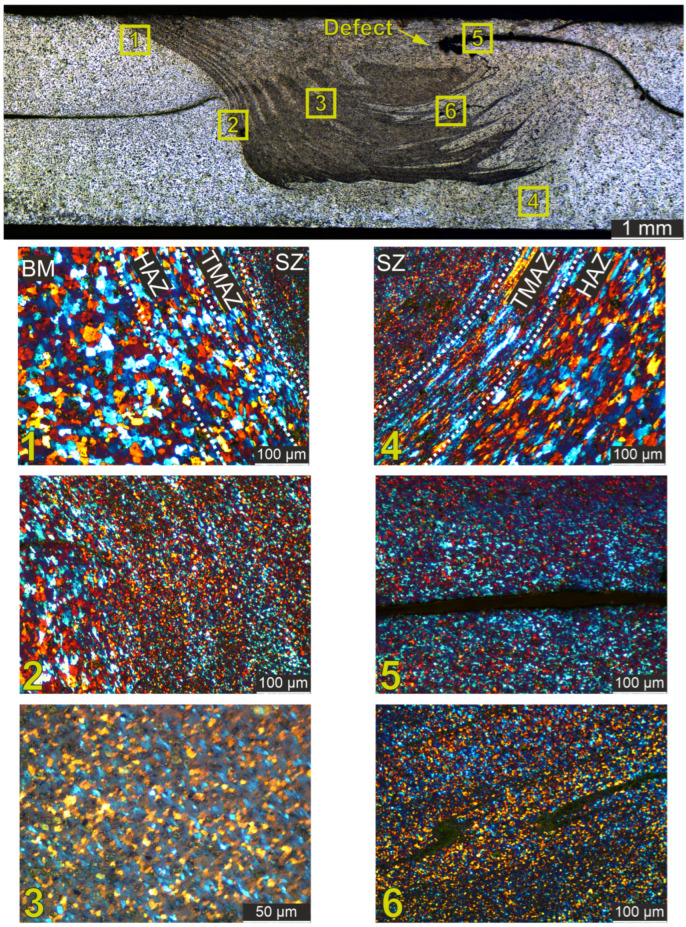
Macro and microstructure analysis of FSW sample. The colorful points represent different crystallographic orientations of the material’s grains.

Macro and microstructure analyses were performed on the specimen to further validate the quality of the weld (Figure 13). The image provided illustrates these analyses, with six different regions marked and examined in detail. The specimen has been electropolished to reveal its microstructural features, and the image shows a cross-section of the FSW joint.

- Region 1 (Base Metal (BM), Heat-Affected Zone (HAZ), Thermomechanically Affected Zone (TMAZ), and Stir Zone (SZ)). This region shows the transition from the BM through the HAZ and the TMAZ into the SZ. The microstructure shows a gradual refinement of the grains from the BM to the SZ, indicating effective thermal and mechanical processing during FSW. The distinct zones highlight the gradient of thermal and mechanical effects on the material [51].
- Region 2 (TMAZ, HAZ): Similar to Region 1, this region provides a detailed view of the microstructural changes within the TMAZ and the HAZ. Grain refinement is evident as the material moves toward the stir zone, showing the progressive effect of the welding process on the material structure. The shape of the grains is a direct result of the compression process, which flattens them into small fractions and causes further grain refinement. A similar evolution of the microstructure was shown in the work of Orlowska et al. [52].
- Region 3 (SZ): The stir zone exhibits a uniform and refined grain structure, indicating effective material mixing and recrystallization during the welding process. This region confirms the high quality of the stir zone, which is critical to the integrity and strength of the weld.
- Region 4 (SZ, TMAZ, HAZ): This region illustrates the microstructural characteristics at the interface between the stir zone (SZ), thermomechanically affected zone (TMAZ), and heat-affected zone (HAZ). The boundaries are well defined and demonstrate the effectiveness of the welding parameters in producing a strong joint with distinct zones that contribute to the overall mechanical properties of the weld.
- Region 5 (SZ—Hooking): This section shows a hooking defect within the SZ. The hooking defect is characterized by a curved, hook-like shape at the interface between the joined materials. [53]. Despite the presence of this defect, the overall grain structure remains consistent with the expected characteristics of a properly welded stir zone. The hooking defect is identified during mechanical testing as a potential crack initiation site that can compromise the structural integrity of the weld.
- Region 6 (SZ—Material Flow Lines): The microstructure in this region shows material flow lines within the stir zone (SZ). The visible lines are likely to flow lines of the material, with changes in shading possibly reflecting the presence of “onion rings” that are characteristic of FSW. These features indicate effective stirring and mixing of the material without the presence of cracks, confirming the overall quality of the weld in this region [54].

**Figure 14:**
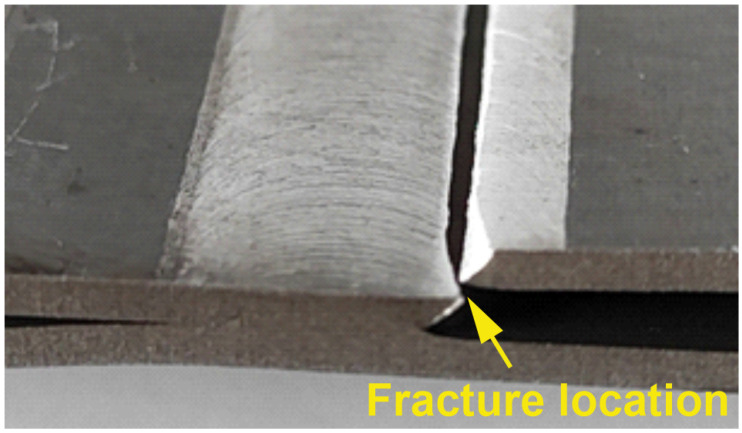
FSW sample after tensile test.

Figure 14 presents the view of the specimen after failure, showing crack initiation and propagation through the identified flaw. The confirmation test validates the effectiveness of the optimized parameters by achieving the predicted load capacities and demonstrating robust weld quality through detailed macro- and microstructural examination. The consistency between predicted and actual performance underscores the reliability of the optimization process used in this study. The observed macrostructure and microstructure are typical of AA2024-T3 alloys, as reported in the publication by Myśliwiec et al. [55], which discusses the butt welding of thin AA2024 sheets. The application of advanced optimization methods for FSW process parameters using commercial software, such as Design Expert 12, has been successfully demonstrated in the studies of Myśliwiec et al. [56].

### 6.2. The Microhardness Analysis

**Figure 15:**
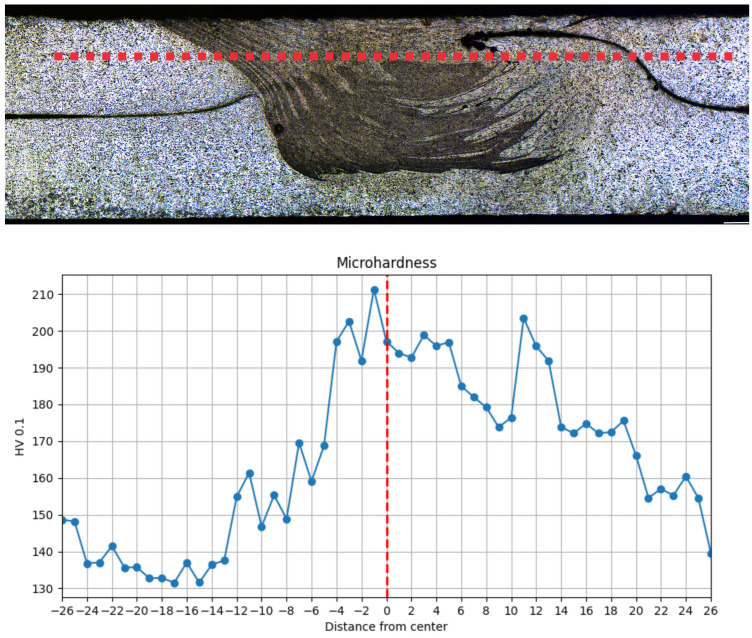
Distribution of microhardness for FSW joint.

The next step in evaluating the formed lap FSW joint was to measure the Vickers microhardness in the cross-section. The measurement method and results are shown in Figure 15. The microhardness profile of the lap FSW joint for the AA2024-T3 alloy shows the following key features: the microhardness of the parent material is at 130–140 HV, increasing the temperature in the heat-affected zone causes a gradual increase in microhardness to a value of 160–170 HV. The peak microhardness (210 HV) in the center of the weld indicates that the refined grains in the weld nugget (the mixing zone) have a higher hardness and, therefore, higher strength than the base material. The change in microhardness in the weld nugget is significant, which is typical for FSW joints of 2024-T3 alloy, but we mostly observe a local decrease in microhardness in this region caused mainly by the dissolution of AlMgCu reinforcing phase particles due to high temperatures [57]. Another factor in the decrease of microhardness is also the movement of dislocations due to intense mechanical deformation [58]. However, in this particular case, we observe the phenomenon of a significant increase in microhardness in the weld nugget. On the one hand, the mechanism of grain reduction due to intense plastic deformation is responsible for this. According to the Hall-Pecha relationship, grain reduction results in increased hardness and strength [59]. Another phenomenon is probably due to the high welding speed and lower temperature in the weld nugget by which the strengthening phase was not dissolved [60]. In addition, the overlap configuration causes an increase in the cross-section of the weld, which leads to better cooling and heat transfer to the environment and tooling. This results in higher longitudinal stresses in the joint, as shown in Staron et al. [61]. The appearance of these stresses can have a negative effect on fatigue and crack propagation in this area. The result can be the appearance of a hook defect in the weld.

## 7. Discussion

The findings of this study provide a comprehensive understanding of the influence of spindle speed and welding speed on the mechanical performance and microstructural characteristics of lap friction stir welded (FSW) joints in AA2024-T3 aluminum alloy. The results clearly demonstrate the nonlinear effects of process parameters on the maximum tensile load (MTL), with significant interaction terms identified through the fifth-order polynomial regression model. Such complexity in parameter interaction has been similarly noted in other recent works applying advanced modeling to FSW optimization [62,63,64]. This highlights that achieving optimal conditions requires not only controlling individual factors but also understanding their combined effects, a challenge addressed increasingly by data-driven and hybrid optimization methods [65,66].

The macro- and microstructural analyses confirm that the optimized parameters effectively refine the grain structure and produce defect-free regions essential for high tensile strength. Grain refinement and improved joint properties following optimized parameter selection have been reported previously, supporting the influence of proper thermal and mechanical conditions on recrystallization kinetics [67,68]. Nevertheless, the hooking defect observed here, often seen in lap FSW configurations, remains problematic. Recent studies have highlighted how hooking can serve as a stress concentrator and limit the joint’s mechanical integrity [69,70]. This defect arises from complex material flow patterns and tool geometry factors, including insufficient plunge depth or inappropriate tool tilt angle, which disturb uniform material flow [53]. The interplay of tool design, heat input, and material plasticity in creating or mitigating hooking defects has become a focal point in recent investigations aiming to reduce defect prevalence [71,72].

In addition, the microhardness analysis revealing increased hardness up to 210 HV in the stir zone is consistent with work showing that refined grain structures and controlled thermal cycles enhance mechanical properties [73]. Minor hardness variations, particularly near the TMAZ, may indicate localized thermal gradients and partial dissolution of strengthening precipitates, aligning with reported thermal effects in similarly optimized FSW joints [63,67].

The use of polynomial regression coupled with a hill-climbing algorithm successfully identified optimal parameters (spindle speed = 1100 rpm and welding speed = 332 mm/min) that maximize joint strength. Confirmation tests validated these optimal conditions, producing MTL values in good agreement with model predictions and corroborating the efficacy of integrating statistical modeling with iterative optimization for FSW parameter tuning. Such approaches reduce reliance on extensive trial-and-error experimentation and can lead to accelerated development cycles and more reliable welding protocols.

Limitations include the controlled laboratory conditions that may differ from industrial settings where factors like tool wear, material batch variations, and dynamic temperature profiles can affect performance. Future work could integrate real-time monitoring and advanced numerical simulations, such as coupled thermo-mechanical finite element models or machine learning-driven adaptive control systems, to enhance understanding and predictability of material flow and defect formation under variable conditions. Expanding the parameter search space and employing global optimization algorithms or evolutionary strategies could further improve weld quality and robustness.

## 8. Conclusions

- The findings of the conducted research led to the following conclusions:
- A fifth-degree polynomial regression model was developed to predict the maximum tensile load (MTL) of friction stir welded (FSW) lap joints, achieving high predictive accuracy with minimal overfitting.
- The experimental results demonstrated a range of MTL values, from 1912 N to 15,336 N, across the tested ranges of spindle speed and welding speed.
- The hill-climbing optimization algorithm identified the optimal welding parameters, which were a spindle speed of 1100 rpm and a welding speed of 332 mm/min, resulting in an MTL of 16,852 N.
- The results of the response surface analysis corroborate the significant interaction between spindle speed and welding speed, delineating regions of maximum MTL values.
- The confirmation tests served to validate the optimized parameters, which were shown to achieve high load capacities and to demonstrate robust weld quality through macro- and microstructural analyses.
- The microhardness profile revealed a peak hardness of approximately 210 HV in the weld center, which can be attributed to grain refinement and the absence of phase dissolution. This indicates that the joint exhibits superior strength.

## Figures and Tables

**Table 1:** Geometric parameters of the FSW tool and welding conditions [22].

Tool Parameters	Value	Tool View
Shoulder diameter D [mm]	12	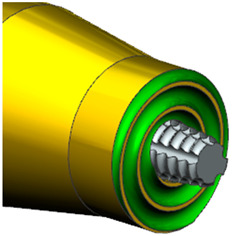
Pin diameter d [mm]	4.5	
Pin height [mm]	2.55	
Tool offset [mm]	0.05	
Dwell time [s]	10	
Tool tilt angle	0°	
Tool plunge speed [mm/min]	2	
Shoulder profile	Flat with spiral groove	
Pin profile	Conical threaded	
D/d ratio of the tool	2.7	
Tool material	H13 Steel	

**Table 2:** FSW process parameters: spindle speed (A) and welding speed (B) with their ranges, coded values, averages, and standard deviations.

Factor	Name	Units	Type	Min.	Max.	Coded Low	Coded High	Mean	Std. Dev.
A	Spindle Speed	rpm	Numeric	600	2200	−1 ↔ 600	+1 ↔ 2200	1400	536.92
B	Welding Speed	mm/min	Numeric	100	350	−1 ↔ 100	+1 ↔ 350	226.79	87.49

**Table 3:** Measured joint maximum tensile load (MTL) in the FSW experiment, including the number of observations, range of values, mean, and standard deviation.

Response	Name	Units	Observations	Min.	Max.	Mean	Std. Dev.	Ratio
R1	MTL	N	112	1912	15,336	6451.97	3071.58	8.02

**Table 4:** Fit summary for polynomial regression models applied to MTL data.

Source	Sequential *p*-Value	Lack of Fit *p*-Value	Adjusted R^2^	Predicted R^2^	
Linear	<0.0001	<0.0001	0.3877	0.3629	
2FI	0.1962	<0.0001	0.3916	0.3541	
Quadratic	0.1153	<0.0001	0.4049	0.3573	
Cubic	<0.0001	<0.0001	0.5213	0.4544	
Quartic	<0.0001	<0.0001	0.8435	0.8234	
Fifth	<0.0001	<0.0001	0.9489	0.9390	Suggested
Sixth	<0.0001	<0.0001	0.9752	0.9683	Aliased

**Table 5:** Assessing the quality of the experimental model. * For a standard deviation of 1.

Term	Standard Error *	VIF	R_i_^2^	Power
A	0.1416	1.00309	0.0031	99.9%
B	0.1357	1.00182	0.0018	99.9%
AB	0.1964	1.00309	0.0031	99.9%
A^2^	0.2453	1.01148	0.0114	99.9%
B^2^	0.2349	1.00965	0.0096	99.9%

**Table 6:** ANOVA for fifth-degree polynomial model applied to MTL data.

Source	Sum of Squares	df	Mean Square	F-Value	*p*-Value	
Model	1.003 × 10^9^	20	5.017 × 10^7^	104.12	<0.0001	significant
A—Spindle Speed	3.854 × 10^7^	1	3.854 × 10^7^	79.99	<0.0001	
B—Welding Speed	7.292 × 10^5^	1	7.292 × 10^5^	1.51	0.2218	
AB	1.535 × 10^8^	1	1.535 × 10^8^	318.49	<0.0001	
A^2^	1.946 × 10^7^	1	1.946 × 10^7^	40.40	<0.0001	
B^2^	2.012 × 10^7^	1	2.012 × 10^7^	41.77	<0.0001	
A^2^B	1.090 × 10^6^	1	1.090 × 10^6^	2.26	0.1360	
AB^2^	4.119 × 10^7^	1	4.119 × 10^7^	85.49	<0.0001	
A^3^	4.509 × 10^7^	1	4.509 × 10^7^	93.59	<0.0001	
B^3^	1.284 × 10^7^	1	1.284 × 10^7^	26.65	<0.0001	
A^2^B^2^	3.609 × 10^7^	1	3.609 × 10^7^	74.91	<0.0001	
A^3^B	1.930 × 10^8^	1	1.930 × 10^8^	400.65	<0.0001	
AB^3^	5.528 × 10^6^	1	5.528 × 10^6^	11.47	0.0010	
A^4^	2.486 × 10^7^	1	2.486 × 10^7^	51.60	<0.0001	
B^4^	8.687 × 10^6^	1	8.687 × 10^6^	18.03	<0.0001	
A^3^B^2^	1.317 × 10^7^	1	1.317 × 10^7^	27.33	<0.0001	
A^2^B^3^	5.118 × 10^4^	1	5.118 × 10^4^	0.1061	0.7454	
A^4^B	5.018 × 10^4^	1	5.018 × 10^4^	0.1041	0.7477	
AB^4^	2.440 × 10^7^	1	2.440 × 10^7^	50.64	<0.0001	
A^5^	4.439 × 10^7^	1	4.439 × 10^7^	92.12	<0.0001	
B^5^	1.765 × 10^7^	1	1.765 × 10^7^	36.62	<0.0001	
Residual	4.385 × 10^7^	91	4.818 × 10^5^			
Lack of Fit	3.533 × 10^7^	7	5.048 × 10^6^	49.80	<0.0001	significant
Pure Error	8.514 × 10^6^	84	1.014 × 10^5^			
Cor Total	1.047 × 10^9^	111				

**Table 7:** Fit statistics for the fifth-degree polynomial model applied to MTL data.

Std. Dev.	694.15	R^2^	0.9581
Mean	6451.97	Adjusted R^2^	0.9489
C.V. %	10.76	Predicted R^2^	0.9390
		Adeq Precision	40.7155

**Table 8:** Final regression equation for predicting MTL in the FSW process.

MTL	=
+2.65761 × 10^5^	
−816.24439	Spindle Speed
−1633.38459	Welding Speed
−2.37849	Spindle Speed· Welding Speed
+1.42349	Spindle Speed^2^
+24.68195	Welding Speed^2^
+0.000460	Spindle Speed^2^· Welding Speed
+0.016034	Spindle Speed· Welding Speed^2^
−0.001068	Spindle Speed^3^
−0.158386	Welding Speed^3^
−2.71047 × 10^−6^	$\mathrm{Spindle}\;{\mathrm{Speed}}^{2}\cdot$ Welding Speed^2^
−4.74309 × 10^−8^	$\mathrm{Spindle}\;{\mathrm{Speed}}^{3}\cdot$ Welding Speed
−0.000039	Spindle Speed· Welding Speed^3^
+3.80250 × 10^−7^	Spindle Speed^4^
+0.000442	Welding Speed^4^
+5.20073 × 10^−10^	$\mathrm{Spindle}\;{\mathrm{Speed}}^{3}\cdot$ Welding Speed^2^
+2.29939 × 10^−10^	$\mathrm{Spindle}\;{\mathrm{Speed}}^{2}\cdot$ Welding Speed^3^
−7.12925 × 10^−12^	$\mathrm{Spindle}\;{\mathrm{Speed}}^{4}\cdot$ Welding Speed
+4.38557 × 10^−8^	Spindle Speed· Welding Speed^4^
−5.22195 × 10^−11^	Spindle Speed^5^
−4.58801 × 10^−7^	Welding Speed^5^

**Table 9:** Experimental and predicted maximum tensile load (MTL) values along with diagnostic metrics for the fifth-degree polynomial regression model.

Run Order	Rotational Speed [rpm]	Welding Speed [mm/min]	Actual Value of MTL [N]	Predicted Value of MTL [N]	Residual	Leverage	Internally Studentized Residuals	Externally Studentized Residuals	Cook’s Distance	Influence on Fitted Value DFFITS
1	600	100	7165.00	7288.17	−123.17	0.248	−0.205	−0.204	0.001	−0.117
2			7299.00	7288.17	10.83	0.248	0.018	0.018	0.000	0.010
3			7057.00	7288.17	−231.17	0.248	−0.384	−0.382	0.002	−0.219
4			7329.00	7288.17	40.83	0.248	0.068	0.067	0.000	0.039
5	800	150	2754.00	3364.21	−610.21	0.213	−0.991	−0.991	0.013	−0.516
6			2800.00	3364.21	−564.21	0.213	−0.916	−0.916	0.011	−0.477
7			2671.00	3364.21	−693.21	0.213	−1.126	−1.128	0.016	−0.588
8			2974.00	3364.21	−390.21	0.213	−0.634	−0.632	0.005	−0.329
9	1000	100	4746.00	4397.76	348.24	0.218	0.567	0.565	0.004	0.299
10			4885.00	4397.76	487.24	0.218	0.794	0.792	0.008	0.419
11			5084.00	4397.76	686.24	0.218	1.118	1.120	0.017	0.592
12			5272.00	4397.76	874.24	0.218	1.425	1.433	0.027	0.757
13	1000	200	6138.00	5629.89	508.11	0.139	0.789	0.787	0.005	0.316
14			5938.00	5629.89	308.11	0.139	0.478	0.476	0.002	0.191
15			6264.00	5629.89	634.11	0.139	0.984	0.984	0.007	0.395
16			6017.00	5629.89	387.11	0.139	0.601	0.599	0.003	0.241
17	1000	300	13,477.00	13,585.93	−108.93	0.141	−0.169	−0.168	0.000	−0.068
18			13,438.00	13,585.93	−147.93	0.141	−0.230	−0.229	0.000	−0.093
19			13,602.00	13,585.93	16.07	0.141	0.025	0.025	0.000	0.010
20			13,511.00	13,585.93	−74.93	0.141	−0.116	−0.116	0.000	−0.047
21	1200	150	5692.00	5464.73	227.27	0.141	0.353	0.352	0.001	0.143
22			5538.00	5464.73	73.27	0.141	0.114	0.113	0.000	0.046
23			5462.00	5464.73	−2.73	0.141	−0.004	−0.004	0.000	−0.002
24			5206.00	5464.73	−258.73	0.141	−0.402	−0.400	0.001	−0.162
25	1200	250	7612.00	8243.00	−631.00	0.128	−0.973	−0.973	0.007	−0.373
26			7547.00	8243.00	−696.00	0.128	−1.074	−1.075	0.008	−0.411
27			7803.00	8243.00	−440.00	0.128	−0.679	−0.677	0.003	−0.259
28			7735.00	8243.00	−508.00	0.128	−0.784	−0.782	0.004	−0.299
29	1400	100	4984.00	5902.63	−918.63	0.173	−1.455	−1.464	0.021	−0.669
30			5059.00	5902.63	−843.63	0.173	−1.336	−1.342	0.018	−0.613
31			4939.00	5902.63	−963.63	0.173	−1.526	−1.538	0.023	−0.702
32			5326.00	5902.63	−576.63	0.173	−0.913	−0.912	0.008	−0.417
33	1400	200	5560.00	6117.56	−557.56	0.106	−0.850	−0.848	0.004	−0.292
34			5602.00	6117.56	−515.56	0.106	−0.786	−0.784	0.003	−0.270
35			5714.00	6117.56	−403.56	0.106	−0.615	−0.613	0.002	−0.211
36			5623.00	6117.56	−494.56	0.106	−0.754	−0.752	0.003	−0.259
37	1400	300	12,271.00	10,669.27	1601.73	0.131	2.475	2.549	0.044	0.989
38			12,121.00	10,669.27	1451.73	0.131	2.243	2.295	0.036	0.891
39			12,189.00	10,669.27	1519.73	0.131	2.348	2.410	0.040	0.935
40			11,405.00	10,669.27	735.73	0.131	1.137	1.139	0.009	0.442
41	1400	350	11,064.00	11,140.53	−76.53	0.191	−0.123	−0.122	0.000	−0.059
42			9862.00	11,140.53	−1278.53	0.191	−2.047	−2.084	0.047	−1.011
43			9916.00	11,140.53	−1224.53	0.191	−1.961	−1.992	0.043	−0.967
44			10,018.00	11,140.53	−1122.53	0.191	−1.797	−1.820	0.036	−0.883
45	1600	150	4556.00	3732.42	823.58	0.141	1.280	1.285	0.013	0.521
46			4525.00	3732.42	792.58	0.141	1.232	1.236	0.012	0.501
47			4989.00	3732.42	1256.58	0.141	1.953	1.985	0.030	0.805
48			4734.00	3732.42	1001.58	0.141	1.557	1.569	0.019	0.636
49	1600	250	5028.00	4620.71	407.29	0.128	0.628	0.626	0.003	0.240
50			4914.00	4620.71	293.29	0.128	0.452	0.450	0.001	0.172
51			4870.00	4620.71	249.29	0.128	0.385	0.383	0.001	0.147
52			4966.00	4620.71	345.29	0.128	0.533	0.531	0.002	0.203
53	1800	100	5092.00	4625.74	466.26	0.218	0.760	0.758	0.008	0.401
54			4889.00	4625.74	263.26	0.218	0.429	0.427	0.002	0.226
55			5102.00	4625.74	476.26	0.218	0.776	0.774	0.008	0.409
56			4612.00	4625.74	−13.74	0.218	−0.022	−0.022	0.000	−0.012
57	1800	200	4465.00	4913.36	−448.36	0.139	−0.696	−0.694	0.004	−0.279
58			4357.00	4913.36	−556.36	0.139	−0.864	−0.863	0.006	−0.347
59			4599.00	4913.36	−314.36	0.139	−0.488	−0.486	0.002	−0.195
60			4741.00	4913.36	−172.36	0.139	−0.268	−0.266	0.001	−0.107
61	1800	300	3250.00	4666.11	−1416.11	0.141	−2.201	−2.250	0.038	−0.912
62			3612.00	4666.11	−1054.11	0.141	−1.638	−1.654	0.021	−0.670
63			3341.00	4666.11	−1325.11	0.141	−2.060	−2.098	0.033	−0.850
64			3493.00	4666.11	−1173.11	0.141	−1.823	−1.847	0.026	−0.749
65	1800	350	4472.00	3473.27	998.73	0.202	1.611	1.625	0.031	0.818
66			3922.00	3473.27	448.73	0.202	0.724	0.722	0.006	0.363
67			3816.00	3473.27	342.73	0.202	0.553	0.551	0.004	0.277
68			4715.00	3473.27	1241.73	0.202	2.003	2.037	0.048	1.025
69	2000	150	3611.00	4131.38	−520.38	0.213	−0.845	−0.844	0.009	−0.440
70			3511.00	4131.38	−620.38	0.213	−1.008	−1.008	0.013	−0.525
71			3761.00	4131.38	−370.38	0.213	−0.602	−0.600	0.005	−0.312
72			3987.00	4131.38	−144.38	0.213	−0.235	−0.233	0.001	−0.122
73	2000	250	5235.00	5184.34	50.66	0.194	0.081	0.081	0.000	0.040
74			4987.00	5184.34	−197.34	0.194	−0.317	−0.315	0.001	−0.154
75			6166.00	5184.34	981.66	0.194	1.575	1.588	0.028	0.778
76			6125.00	5184.34	940.66	0.194	1.509	1.520	0.026	0.745
77	2200	300	5828.00	5831.36	−3.36	0.224	−0.006	−0.005	0.000	−0.003
78			5552.00	5831.36	−279.36	0.224	−0.457	−0.455	0.003	−0.245
79			6836.00	5831.36	1004.64	0.224	1.643	1.659	0.037	0.892
80			5555.00	5831.36	−276.36	0.224	−0.452	−0.450	0.003	−0.242
81	2200	350	5228.00	6187.20	−959.20	0.242	−1.587	−1.601	0.038	−0.905
82			5928.00	6187.20	−259.20	0.242	−0.429	−0.427	0.003	−0.241
83			6865.00	6187.20	677.80	0.242	1.122	1.123	0.019	0.635
84			6133.00	6187.20	−54.20	0.242	−0.090	−0.089	0.000	−0.050
85	2200	200	5738.00	5860.18	−122.18	0.234	−0.201	−0.200	0.001	−0.111
86			5759.00	5860.18	−101.18	0.234	−0.167	−0.166	0.000	−0.092
87			6288.00	5860.18	427.82	0.234	0.704	0.702	0.007	0.388
88			5890.00	5860.18	29.82	0.234	0.049	0.049	0.000	0.027
89	2200	100	1912.00	1992.21	−80.21	0.248	−0.133	−0.133	0.000	−0.076
90			2065.00	1992.21	72.79	0.248	0.121	0.120	0.000	0.069
91			2058.00	1992.21	65.79	0.248	0.109	0.109	0.000	0.062
92			1951.00	1992.21	−41.21	0.248	−0.068	−0.068	0.000	−0.039
93	600	300	10,620.99	10,887.32	−266.33	0.224	−0.436	−0.434	0.003	−0.233
94			10,933.00	10,887.32	45.68	0.224	0.075	0.074	0.000	0.040
95			10,641.00	10,887.32	−246.32	0.224	−0.403	−0.401	0.002	−0.216
96			10,884.00	10,887.32	−3.32	0.224	−0.005	−0.005	0.000	−0.003
97	600	200	8110.00	7771.51	338.49	0.234	0.557	0.555	0.005	0.307
98			8014.00	7771.51	242.49	0.234	0.399	0.397	0.002	0.220
99			7917.00	7771.51	145.49	0.234	0.239	0.238	0.001	0.132
100			8436.00	7771.51	664.49	0.234	1.094	1.095	0.017	0.606
101	800	250	5529.00	5453.20	75.80	0.194	0.122	0.121	0.000	0.059
102			5168.00	5453.20	−285.20	0.194	−0.458	−0.456	0.002	−0.223
103			5127.00	5453.20	−326.20	0.194	−0.523	−0.521	0.003	−0.255
104			5193.00	5453.20	−260.20	0.194	−0.417	−0.416	0.002	−0.204
105	1000	350	15,336.00	14,230.29	1105.71	0.202	1.783	1.805	0.038	0.908
106			14,590.00	14,230.29	359.71	0.202	0.580	0.578	0.004	0.291
107			14,274.97	14,230.29	44.68	0.202	0.072	0.072	0.000	0.036
108			13,972.00	14,230.29	−258.29	0.202	−0.417	−0.415	0.002	−0.209
109	600	350	5009.00	5290.96	−281.96	0.242	−0.467	−0.465	0.003	−0.263
110			5514.00	5290.96	223.04	0.242	0.369	0.367	0.002	0.208
111			5435.00	5290.96	144.04	0.242	0.238	0.237	0.001	0.134
112			5219.00	5290.96	−71.96	0.242	−0.119	−0.118	0.000	−0.067

## Data Availability

The original contributions presented in this study are included in the article. Further inquiries can be directed to the corresponding author.
